# Dichotic sentence identification test in Portuguese: a study in young adults

**DOI:** 10.1016/j.bjorl.2020.11.018

**Published:** 2021-01-05

**Authors:** Maristela Julio Costa, Sinéia Neujahr dos Santos, Eliane Schochat

**Affiliations:** a Universidade Federal de Santa Maria (UFSM), Programa de Pós-Graduação em Distúrbios da Comunicação Humana, Santa Maria, RS, Brazil; b Universidade Federal de Santa Maria (UFSM), Hospital Universitário de Santa Maria, Programa de Pós-Graduação em Distúrbios da Comunicação Humana, Santa Maria, RS, Brazil; c Universidade de São Paulo (USP), Faculdade de Medicina, Departamento de Fisioterapia, Fonoaudiologia e Terapia Ocupacional, São Paulo, SP, Brazil

**Keywords:** Auditory perception, Hearing disorders, Hearing tests

## Abstract

**Introduction:**

Among the currently-applied auditory processing tests, dichotic listening tests have been widely used, since they allow investigating the hemispheric and inter-hemispheric function and their respective skills to process the received auditory information.

**Objective:**

To obtain normality reference measures with the new dichotic sentence identification test in right-handed adults with normal hearing.

**Methods:**

Quantitative, observational, cross-sectional study. 72 subjects were assessed, aged 19–44 years, right-handed, with normal hearing, without hearing complaints. The dichotic sentence identification test consists of different lists of sentences, which were combined two by two and presented at the same time, using earphones in both ears, at 50 dB Sensation Level. The test was applied in four stages: training of the 3 stages, free attention, right and left directed attention, thus evaluating different auditory skills.

**Results:**

In the free attention task, the average percentage of correct answers in the right ear was 93.59% and in the left ear 86.06%, with a statistically significant difference between the ears, with an advantage for the right ear. In the directed attention task, the average percentage of correct answers was 99.37% in the right ear and 98.8% in the left ear, with no statistical difference between the ears.

**Conclusion:**

It is suggested, as a normality reference for the stage of free attention, 90%–100% for correct answers for the right ear and for the left ear, from 80% to 100%. When there is asymmetry between the ears, differences of up to 20% are expected, with an advantage for the right ear For the directed attention stage, the expected normality reference values ​​are 100% for the right ear and for the left ear, with no asymmetry between the ears; however, if it occurs, a difference of 10% is expected between the ears, with an advantage for the right ear.

## Introduction

Behavioral tests are among the innumerable possibilities for investigating hearing, providing information about the individual's abilities to process the received auditory information.^1^, ^2^, ^3^, ^4^

Considering that the physiological processes involved in auditory processing are still not fully understood,^5^ the behavioral assessment is considered a very important tool to help in the diagnosis of auditory processing disorders.^6^, ^7^, ^8^

Among the several currently available auditory processing tests,^3^, ^9^, ^10^, ^11^ the dichotic listening tests have been widely used, as they provide information on hemispheric and inter-hemispheric functions, since their application allows investigating clinical hearing disorders and conditions that reveal abnormal hemispheric function, involving the abilities of binaural integration and separation, as well as the individuals' attention, memory and executive function impairment.^3^, ^4^, ^12^, ^13^

Since the 1950s, there have been references to dichotic listening, which has been defined as “a simultaneous stimulation of both ears, but with different stimuli in each ear”,^14^ and there have been study references using such tests with different speech stimuli as early as in the 1950s.^15^

The processes of binaural integration and separation are critical for daily listening, and deficits in these areas can be behaviorally expressed as the difficulty listening in unfavorable listening environments, which justifies and reinforces the importance of such assessment instruments.^6^

Despite the relevance of the information provided by the dichotic listening tests, there are still few tests available in our language, and in Brazil, the DSI test, translated and adapted into Brazilian Portuguese, is the only one that uses sentences as a stimulus,^16^ being minimally affected by peripheral hearing loss^9^, ^16^; however, as they are meaningless sentences and their response strategy is to identify the sentences that have been heard when presented in writing, it limits the application in cases of children or people who do not have functional reading skills or those who have vision problems.

Thus, considering the scarcity of materials in our country that evaluate dichotic listening using sentences as a stimulus, and the importance of the information obtained through them, a new sentence test was developed, with sentences that have meaning, in Brazilian Portuguese, to be dichotically applied (DST).^17^

As this is a new test, for it to be used in clinical routine and research and, considering the countless factors involved in the development and application of tests to assess auditory processing abilities, the present study aimed at obtaining reference measures of normality in adult individuals with normal hearing, for the new dichotic sentence identification test, in Brazilian Portuguese.

## Methods

The study was carried out from October 2016 to February 2017 at the *Laboratório de Investigação Fonoaudiológica em Neuroaudiologia* of the Department of Physical Therapy, Speech Therapy and Occupational Therapy at *Faculdade de Medicina da Universidade de São* Paulo, analyzed and approved by the Research Ethics Committee from *Faculdade de Medicina da Universidade de São Paulo* (CEP-FMUSP) under CAAE number: 59617416.9.1001.0065. The individuals were invited to participate through an invitation sent by email, posters or personal contact in the city of São Paulo and randomly selected by convenience. All individuals who received information on the research procedures and agreed to participate, by signing the free and informed consent form, were evaluated by one of the audiologists responsible for the study and the results were analyzed and discussed with the others.

The inclusion criteria for sample composition were: young adult individuals, aged 19–42 years, right hand preference, native Brazilian Portuguese speakers, with tonal thresholds better than or equal to 25 dB hearing level at the frequencies of 250–8000 Hz, having completed at least Elementary School and showing a dichotic digit test performance ≥95%.^3^

The exclusion criteria were: presenting any factor that could interfere with the adequate performance of the test, such as evident neurological, psychological and phonological alterations, middle ear or general health problems.

Regarding the level of schooling, they were classified into three categories: category 1 – complete junior high, complete or incomplete high school and incomplete college/university; category 2 – complete college/university, residents, ongoing postgraduate studies and those with a master's degree; category 3 – ongoing doctoral degree, doctoral degree (Ph.D.) and post-doctoral degree.

Initially, the participants answered a directed anamnesis, with questions regarding personal data, hand preference, level of schooling, otological history, hearing complaints; thereafter, a visual inspection of the external auditory canal was performed, so that the assessment itself could be initiated.

At the first stage, hearing thresholds were obtained by air conduction at the frequencies of 250 to 8000 Hz. Then, the Dichotic Digit Test (DDT) was applied,^3^, ^18^ evaluating only the free attention task and, finally, the Dichotic Sentence Identification (DSI) test was applied.

The evaluations were carried out in an acoustically treated room, using a Grason-Stadler Audiometer, model GSI-61, and earphones model TDH 50. The DDT and DSI tests were presented digitally recorded in the WAV format, using a tablet attached to the audiometer.

The sentences that originated the DSI test were previously developed and published and are part of a test consisting of seven sentence lists developed in the Brazilian Portuguese Language (LSP-BR).^19^, ^20^ The lists consist of 10 affirmative sentences, different, but equivalent,^21^ containing 4–7 words, which represent common everyday conversational situations, and are phonetically balanced.

After the development of this material, different studies have been carried out, with different sample groups, confirming its applicability and determining validation measures, and it has been verified that they can be applied to children from 7 years of age,^22^ as well as adults^19^ and the elderly.^23^

Based on that, the abovementioned lists of sentences were adapted and then presented in a previously published dichotic version,^17^ which resulted in a protocol containing 8 different combinations of lists forming pairs of sentences, using their duration as the main criterion.

The protocol consisted of two options of combined sentence lists, called sequences 1 and 2, with 4 combinations of different lists in each sequence, to be applied in the following order of presentation: training phase, free attention, right directed attention and then left, taking care that no list would be repeated within the same sequence, nor presented in the same position, and also avoiding the simultaneous presentation of similar sentences or words.

This protocol was digitally recorded and presented using a tablet and consisted of nine tracks: Track 1—pure calibration tone and then, the eight tracks with the two different sequences, recorded in independent channels, thus allowing the levels of presentation in each channel to be independently adjusted by ear. The response times established according to the tasks were: for training and free attention, eight seconds and, for the directed attention stage, 4 s.

The material was presented at 50 dB sensation level,^18^ in both ears, above the tritonal average of 500, 1000 and 2000 Hz, with the two channels of the equipment having been calibrated, using the 1000 Hz pure tone, placed at the zero level of the audiometer's VU Meter.

Before starting the application, the subjects were instructed on the evaluation strategy: different sentences would be presented simultaneously¸ in both ears and the type of response in the first task would consist in repeating the sentences heard in both ears (free attention) and in the second and third tasks, the individuals should repeat the sentences heard only in the right ear (RE), then in the left ear (LE) (directed attention).^24^

Initially, the training phase was carried out, to familiarize the assessed subject with each test task in which 3 pairs of sentences were presented and they were asked to repeat the sentences heard in both ears; then, another three pairs of sentences, asking them to repeat the sentences in the RE, then another three pairs, in the LE and three remaining pairs, again requesting the answer from both ears.

The repetition of the free attention task training, in the last three pairs of sentences, was used because this task is the most complex and, therefore, the one that demands the most from the patient.

The correct answer was considered when the patient repeated the entire sentence heard correctly, so when any word, part of the sentence or all of it was not repeated correctly, the error rate of 10% was attributed. Also, for the analysis and interpretation of the results when comparing the performance between the ears, when the result is positive, it means that the RE was better than the LE and when it is negative, the LE was better than the RE.

The two sequences were always presented in the same order, starting with sequence 1 and then sequence 2. In total, 101 individuals were assessed, 29 of which were excluded, and 72 retained in the study.

### Data analysis

The data were analyzed using the SAS® MIXED procedure, version 9.4. The statistical model included the fixed effects of the ear, sequence and interaction between ears and sequences. Patients and residual variance were considered to be random effects. Patients' age, gender and level of schooling were included in the model as covariates. When differences were observed, the means were compared using the LS-means resource and the interactions were broken down when significant at 5% probability. The frequencies of the test responses were calculated for the cutoff points (60; 70; 80; 90 and 100) and the probability of occurrence between the ears and between the cutoff points. The Shapiro-Wilk test was used to analyze the normality of data, and since they did not show a normal distribution, and did not adjust to any transformation, the free attention and directed attention variables were analyzed by the Wilcoxon test. The probability threshold values ​​of 5% for significance and 10% for trend were adopted.

The power analysis of the sample size calculated by the Power procedure, based on Cochran Sampling Techniques, indicated that 72 patients are sufficient, confirmed by the power analysis, with a probability result of 0.99.

## Results

The sample consisted of subjects aged 19–42 years, with a mean age of 28.06 years, of which 56.94% were females and 43.06% males; regarding the level of schooling, 47.22% belonged to Category 1; 33.33% to Category 2 and 19.44% to Category 3.

No significant difference was observed between the measures in the free and directed attention tasks, when comparing the obtained results considering gender, age, level of schooling and the different sequences; the only statistical difference observed was related to ear side (RE and LE) and, therefore, the data were analyzed separately only according to the ear side.

**Figure 1:**
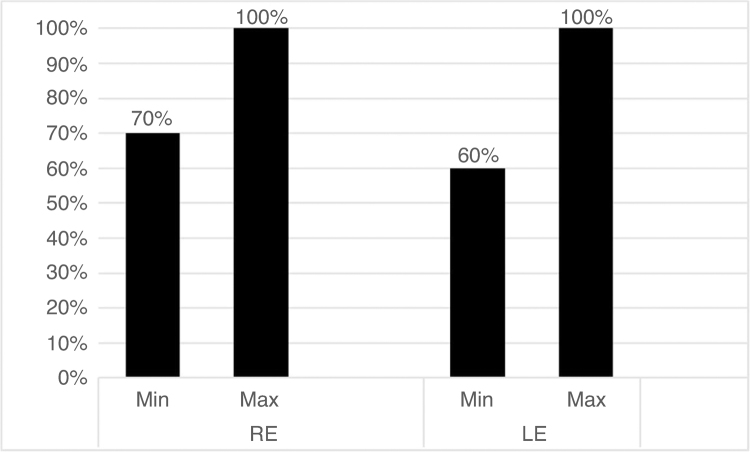
Minimum and maximum performances in the free attention task for the right ear (RE) and left ear (LE).

Initially, the minimum and maximum performances obtained for the free attention task can be observed in Fig. 1.

**Figure 2:**
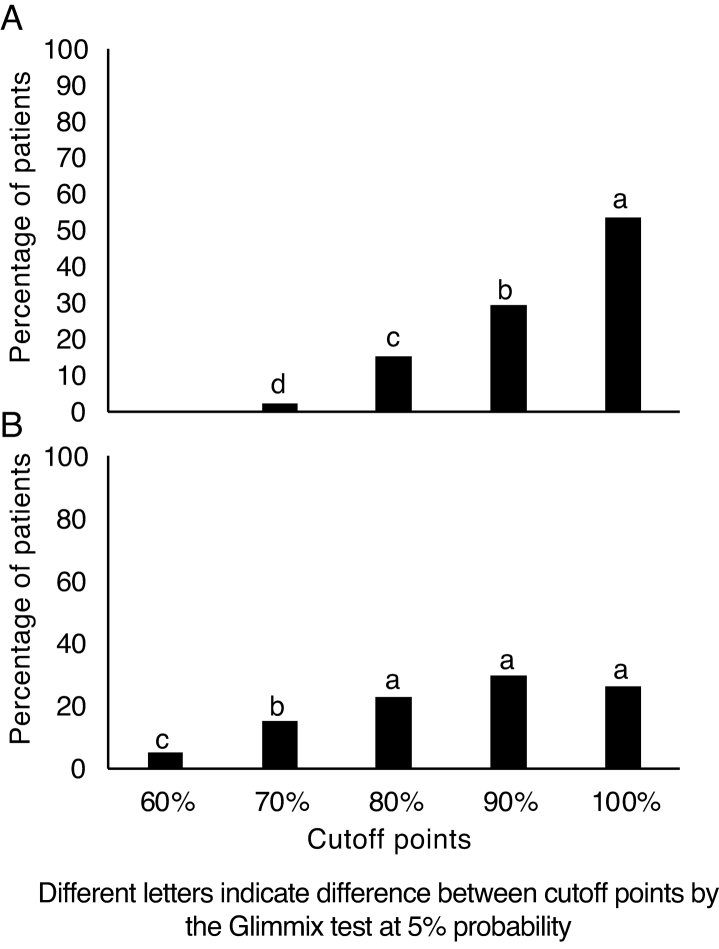
Performance frequency of normal-hearing young adults, in the free attention task, according to the cutoff points for the right (A) and left (B) ears.

Table 1 and Fig. 2 depict the performances of the assessed subjects, considering as cutoff points, and the result of the statistical analysis, which determined the probability of the occurrence of each performance, according to the ear side, for the free attention task.

**Table 1:** Different performances in normal-hearing young adults, considered as cutoff points according to the ear side, for the free attention task and the frequency of their occurrence.

Cutoff point	Ear		SEM	Probability
	Right, n (%)	Left, n (%)		Ear/Cutoff point
60%	0 (0.0d)	4 (4.86c)	0.18	<0,01*
70%	1 (2.09d)	11 (15.28b)		
80%	11 (15.13c)	17 (23.61a)		
90%	21 (29.17b)	21 (29.86a)		
100%	39 (53.48a)	19 (26.39a)		

SEM, standard error of the mean.

ANOVA performed by the Glimmix Test considering 5% for significance *.

Table 2 shows the averages of responses considering the variable side of the ear, in the free attention task and the statistical result, comparing the performance between the ears.

**Table 2:** Averages of the performance of normal-hearing young adults, according to the side of the ear, for the free attention task.

Variable	Ear		SEM	Probability
	Right (SD)	Left (SD)		Ear
Free attention (%)	93.59 (8.12)	86.06 (11.74)	0.83	<0.01*

SEM, standard error of the mean.

ANOVA performed by the Wilcoxon Test, significance *p* < 0.05*.

Table 3 shows the distribution of performance differences obtained between the ears in the free attention task and the frequency of their occurrence.

**Table 3:** Distribution of patients and frequency of occurrence of differences in the performance between the right and left ears in the free attention task.

Difference	(n) % of patients
−20	(2) 2.78
−10	(6) 9.03
0	(24) 32.64
10	(22) 30.55
20	(12) 15.97
30	(6) 7.64

**Figure 3:**
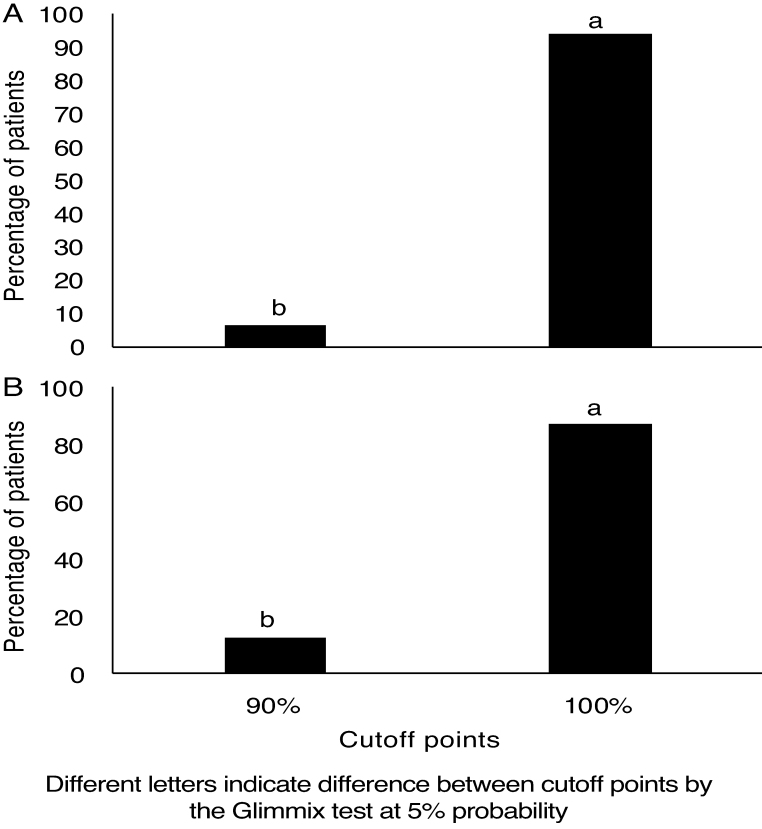
Frequency of patients for the directed attention task according to the cutoff points for the right (A) and left (B) ears.

Table 4 and Fig. 3 show the performance in the directed attention task, considered as cutoff points and the result of the statistical analysis, which determines the probability of the occurrence of each performance according to the ear side.

**Table 4:** Frequency of patients according to the different cutoff points regarding the ear side, for the directed attention task.

Cutoff point	Ear		SEM	Probability
	Right (n)	Left (n)		Ear/ Cutoff point
60%	0,00b (0)	0.00b (0)	0.20	0.04*
70%	0,00b (0)	0.00b (0)		
80%	0,00b (0)	0.00b (0)		
90%	6,25b (4)	12.5b (9)		
100%	93,75a (68)	87.5a (63)		

SEM, Standard Error of the Mean.

ANOVA performed by the Glimmix Test considering 5% for significance * and 10% for trend ^ƭ^.

Table 5 shows the averages of responses considering the ear side variable, in the directed attention task and the statistical result comparing the performance between the ears.

**Table 5:** Averages of the performance of normal-hearing young adults, according to the side of the tested ear, for the directed attention task.

Variable	Ear		SEM	Probability
	Right (SD)	Left (SD)		Ear
Directed attention (%)	99,37 (2,43)	98,80 (3,32)	0,24	0,07

SEM, Standard Error of the Mean.

ANOVA performed by the Wilcoxon Test, significance *p* < 0.05*.

SD, standard deviation.

## Discussion

Based on the descriptive and inferential analysis of the sample subjects’ characteristics, their homogeneity was verified, since there was no effect on the results obtained according to the variables gender, age and level of schooling. These results confirm the sample homogeneity, which was an objective, as it is known that the performance in relation to these tests can be affected by different factors and that the auditory processing skills may be influenced by gender, age, level of schooling, cognitive aspects and audibility.^10^, ^24^

It was also verified that there was an equivalence between the results obtained with the different combinations of sentence lists (sequence 1 and sequence 2), which allows us to affirm that any sequence can be used, without the influence of the chosen sequence on the performance of the individuals to be assessed, emphasizing that it is necessary to apply only one sequence in a typical evaluation.

Continuing the analysis of the results obtained in the free attention task, it was observed that the lowest performance in the right ear was 70% correct answers, whereas the highest was 100%. In the left ear, the minimum response was 60% and the maximum response was 100% (Fig. 1). Thus, these values ​​were defined as cutoff points, aiming to determine the expected performance for this population.

Therefore, in the free attention task for the RE (Table 1 and Fig. 2), considering the different cutoff points, it can be observed that 82.65% of the subjects showed a performance with 90% and 100% of correct answers, and the probability analysis by the frequency of occurrence showed a high probability of occurrence of 100% of correct answers and an average probability of 90%, while for the cutoff points of 80% there was low probability and 70% of minimum probability.

In turn, for the LE, it was found based on the frequency of occurrence in each cutoff point, that 79.86% of the subjects had performances that varied between 80%, 90% and 100%, with the frequency of occurrence at these three cutoff points being similar, and the analysis of the probability of the occurrence frequency showed a high probability of occurring for any of these results, and a low probability of 70% and a minimum probability of 60% of correct answers.

In the literature, there is one study that applied the same test material, in normal hearing adults aged 18–40 years, whose performances were obtained in the free attention task in the RE that ranged from 80% to 100% and in the LE from 70% to 100%.^17^

According to a study with the DSI in the English Language,^16^ reference values ​​were not determined by ear; they only reported that the responses in the free attention task, in normal hearing individuals, varied from 75% to 100%.

In a study with the DSI in Portuguese,^11^ 80% of correct answers in the RE and 60% in the LE were obtained for the age group of 20–29 years old, and for the age groups of 30–39 and 40–49 years old, 70% of correct answers in RE and 60% in LE.

Table 2 shows the average performance values ​​obtained according to the ear side: for the RE, 93.59% and LE, 86.06%, showing that there was a statistically significant difference between the ears, with an advantage for the RE.

Comparing these findings with the reviewed literature, it was verified in a previously mentioned study that used the same evaluation protocol of the present research, an average performance of 93.33% in the right ear and 90.24% in the left ear.^17^

In turn, when using the DSI test in the English language,^16^ the average percentages of correct answers obtained in the RE were 94.2% and in the LE, 93.5%, also indicating an advantage of the LE, although it was not statistically significant.

Additionally, in another study,^11^ which used the DSI test in Portuguese, they obtained average performances in the RE of 93.70% and in the LE, 88.60%.

Therefore, based on the data analyzed here, the following normality reference values ​​are suggested for normal young hearing adults, in the free attention task: performance ≥90% in the RE, and ≥80% in LE, with a variation ±10%, according to the SD.

Comparing the data from the present investigation with those obtained from different studies, variations in extreme values ​​are observed, which are justified by the differences between the tests used, such as language, sentence structure, application strategy and age range; however, the mean values ​​are quite similar and there is a constant result among the different authors, which is the tendency of RE advantage in the free attention task.^7^, ^16^, ^24^, ^25^, ^26^

The advantage of the RE in dichotic tests is expected in right-handed adults with normal hearing, since it is described as a consequence of the preferential conduction of messages received in the RE directly to the left hemisphere, typically dominant for language.^26^

Some potential difficulties can be identified in this task, since the challenges include memory and information processing velocity, and the fact that in the free attention task, it is necessary to monitor both ears to identify the two sentences heard, store them in short-term memory and respond to both sentences, requiring the mobilization of memory, attention and rapid processing resources.^27^

From the anatomical point of view, when the stimulus is verbal, the RE signal is transmitted directly to the left hemisphere of the cortex through dominant contralateral pathways, while the LE signal is sent to the right hemisphere and then to the left hemisphere through the corpus callosum, taking longer for the signal sent by the LE to be perceived, which justifies the advantage of the RE.^7^, ^27^, ^28^

Therefore, continuing the analyses and considering the differences observed in the performance between the ears, it is also important to consider and analyze the amplitude of this difference, since the asymmetry between the ears, although expected, when accentuated may indicate a significant alteration in the auditory processing.^27^, ^29^

We can still mention another study,^12^ which considers as an important limitation of the currently available dichotic tests the fact that many tests do not have standardized measures on the interaural asymmetry index.

When comparing the differences between the ears (Table 3), it can be observed that 79.16% of the subjects showed results that varied from 0% to 20% of difference between the ears, which can be expected as normal for normal-hearing and right-handed young adults, showing symmetry or some degree of asymmetry between the ears, which can be up to 20%, with an advantage for the RE.

Several studies^7^, ^16^, ^24^, ^25^, ^26^ discussed the advantage of the RE in dichotic listening tests, but only one^16^ suggests the expected values ​​for adult individuals, with tonal thresholds of up to 40 dBHL, reporting that the difference between the ears should not exceed 16%.

One study^24^ reported that the interaural asymmetry in the DSI increased with age, varying from 5% at 50 years old to 40% in the age range over 90 years, reporting that the interaural asymmetry in dichotic listening can be attributed to age-related structural mechanisms, involving the maturation and aging processes.

Finally, when analyzing the results obtained in the directed attention task, (Table 4 and Fig. 3), it can be observed that the probability analysis indicated a high probability of performance of 100% and a low probability of 90% in both ears. In turn, when analyzing the averages of performances in the directed attention task (Table 5), it was observed that most individuals had 100% of correct answers both in the RE and LE.

In the investigation carried out with the DSI test in Portuguese,^11^ in the directed attention task, there was no difference between the mean percentage of correct answers obtained for both ears, with an average performance of 98% of correct answers being observed for all ages in the directed attention stage, both in the RE and LE, which, according to the authors, was already expected, as the individuals included in the study did not have auditory processing disorders, demonstrating their ability to direct auditory attention to the proposed stimulus and show a good performance in the directed attention task. The authors suggested reference values ​​of 90% of correct answers for both ears in the age group of 13–39 years, and of 80% in RE and 70% in LE in the age group of 40–49 years.

Therefore, 100% performance for both ears is suggested as an expected value for this population in the directed attention task, and despite a trend for better responses in the RE, there was no significant difference between the ears, indicating that symmetry is expected between the ears in this task, and when there is asymmetry, it should not be >10%, with an advantage for the RE.

In the directed attention task, the cognitive load imposed by the task is considerably attenuated, as only one ear needs to be monitored at a time and, thus, the memory, attention and processing velocity resources are much less requested,^27^ which justifies the better performance in this task, as well as the symmetry between the ears.

In any auditory processing evaluation, it is known how important it is for the objectives to be clear and the evaluator to be attentive to the characteristics and complaints of the individual being evaluated, but this is even more relevant in dichotic listening tests with verbal stimulus, since gender, age, level of schooling and cognition may have a direct influence on the results.^10^, ^24^

Furthermore, the importance of tests that assess binaural auditory processing is reinforced, and the inclusion of binaural tests in the audiological evaluation assessment is encouraged, as their information will contribute to establish alternative forms of audiological rehabilitation.^30^

Finally, it should be noted that the normal values ​​determined in this research were established considering that the test characteristics and the way the results are interpreted contributed to the test sensitivity, since a 10% error was attributed to a word or the entire sentence. Therefore, it is important to highlight the importance of the examiner's clinical perspective during the test performance, and to be very attentive to the individual’s physical and emotional conditions, as they can significantly influence the results. Moreover, to ensure a more accurate diagnosis, always consider the set of information and results from other tests that assess auditory processing, which will be part of the battery of tests selected to be applied to each subject.

## Conclusion

The performance suggested as a normality reference for the new dichotic sentence identification (DSI) test, in Brazilian Portuguese, for right-handed young adults with normal hearing and without hearing complaints, in the different evaluated tasks were: for the free attention task in RE, ≥90% of correct answers and, in the LE, ≥80% of correct answers, and there may be asymmetry between the ears, up to 20%, with an advantage for the RE. For the directed attention task, in the RE and LE, the values ​​are 100% of correct answers, with asymmetry between the ears not being expected.

## Conflicts of interest

The authors declare no conflicts of interest.
